# T.I.M.S: TaqMan Information Management System, tools to organize data flow in a genotyping laboratory

**DOI:** 10.1186/1471-2105-6-246

**Published:** 2005-10-12

**Authors:** Stéphanie Monnier, David G Cox, Tim Albion, Federico Canzian

**Affiliations:** 1 International Agency for Research on Cancer, F-69372, Lyon, France; 2 Department of Epidemiology, Harvard School of Public Health, Boston, MA 02115, USA; 3 Menzies Research Institute, University of Tasmania, Hobart 7001, Australia; 4 German Cancer Research Center (DKFZ), D-69120, Heidelberg, Germany

## Abstract

**Background:**

Single Nucleotide Polymorphism (SNP) genotyping is a major activity in biomedical research. The Taqman technology is one of the most commonly used approaches. It produces large amounts of data that are difficult to process by hand. Laboratories not equipped with a Laboratory Information Management System (LIMS) need tools to organize the data flow.

**Results:**

We propose a package of Visual Basic programs focused on sample management and on the parsing of input and output TaqMan files. The code is written in Visual Basic, embedded in the Microsoft Office package, and it allows anyone to have access to those tools, without any programming skills and with basic computer requirements.

**Conclusion:**

We have created useful tools focused on management of TaqMan genotyping data, a critical issue in genotyping laboratories whithout a more sophisticated and expensive system, such as a LIMS.

## Background

The completion of the human genome sequence has brought a wealth of data on genetic variation, mostly in the form of single nucleotide polymorphisms (SNPs).

As a consequence, SNP genotyping has recently become a major activity for studies of disease susceptibility and pharmacogenetics. While techniques for ultra-high throughput (hundreds of millions of genotypes scored per year) are becoming available, the vast majority of genotyping laboratories around the world are equipped with technology suited for low- to high-throughput (up to a few million genotypes scored per year). The 5' nuclease assay, also known as TaqMan, is one of the main approaches currently used for genotyping [1,2].

**Figure 1:**
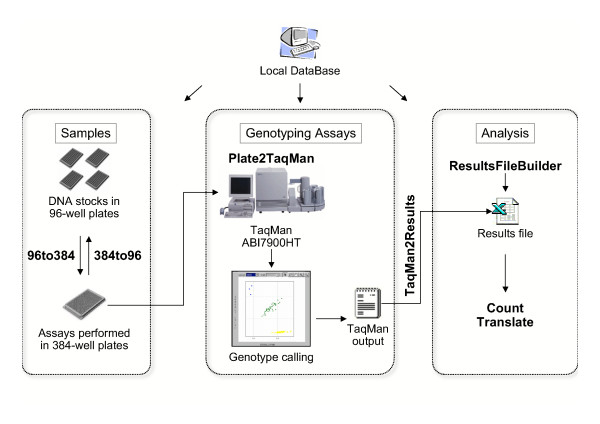
**Data flow in a SNP genotyping laboratory. **Data flow is divided into management of samples, genotyping assays and genotyping results (dotted outlines). In bold, names of macros described in the text. All operations can be interfaced with a local database storing e.g. details about names of samples in plates, sequences of primers and probes used as genotyping assays, genotyping results.

Small genotyping laboratories are rarely equipped with sophisticated Laboratory Information Management Systems (LIMS) to follow the flow of information on samples and genotyping results. In a typical workflow (Figure 1 represents the workflow in our laboratory at the Genome Analysis Team, International Agency for Research on Cancer), many files and many different formats are managed. For example, DNA stocks are usually stored in 96-well plates but genotyped in 384-well plates. Sample preparation and handling are often done with different kind of robots (e.g. instruments for DNA extraction, liquid handling robots for set-up of PCR reactions, plate cranes linked with TaqMan instruments), each one with its own input and output file format.

We have developed a software suite for genotyping laboratories, aimed at improving data workflow, saving time and preventing errors linked to managing data by hand. Previously, other groups have developed tool focusing on management of data workflow [3,4]. Differently from previous solutions, we propose a set of independent tools, with a focus on ease of use. Users can download some of the macros or the whole suite, according to their needs. In addition, previously developed programs were tailored to linkage analysis projects, while TIMS is aimed at genotyping in the context of population-based association studies, and therefore it includes several new functions that allow to treat more easily large numbers of sample plates, and to generate input files for downstream software that is not relevant to linkage analysis projects.

## Implementation

TIMS is a suite of tools written in Visual Basic. Given that genotyping data are usually represented in tabular format, we have chosen to use the version of Visual Basic embedded in Microsoft Excel. This offers the advantage of being available to most computer users, and allows interoperability between Windows and MacOS. The different tools have the format of Visual Basic macros, and they are used at different steps of the workflow (Figure 1):

### 1. Sample processing

We used a simple plate map format to document the localization of DNA samples (one file per plate). Often, both 96-well and 384-well plates are used in a laboratory, which creates the necessity of conversion between the two formats.

Macro "**96to384**" is used to create the map of a 384-well plate, starting from the map of four 96-well plates. Two ways of interleaving sample location from 96-well to 384-well plates are proposed ("Z" or "N", reading from left to right or from top to bottom). The user has to provide a list of files corresponding to 96-well plates to be read by the program and treated in batch. An optional function is offered to the user, to create automatically all the Taqman input files from the newly created 384-well plates. This is the function of the macro "**PlateToTaqMan**", which transforms the plate maps into files that can be directly used as TaqMan input files, in batch for 96-well plate and 384-well plates.

Reciprocally, knowing the composition of a 384-well plate in advance, a user may want to reconstruct the four 96-well DNA source plates. The macro "**384to96**" gives the structure of the 96-well plates to start with, in order to have the required 384-well plate.

### 2. Results management

The program "**ResultsFileBuilder**" builds an Excel file, ready to receive the genotyping results. The format of the result file is compatible with our previously published software [5]. The macro copies the list of samples (including information on plate number and location within plates) from a series of plate map files.

The output of the TaqMan instrument is a text file, with genotype information that has to be parsed to make use of it. Another macro, called "**TaqMan2Results**", is used to transfer the results from the Taqman output file into the results file described above.

Several functions have been built in this macro:

- The possibility to compare analysis of the same assay read by two users. In our laboratory, we routinely perform double blind reading of each TaqMan plate, for quality control. In this case, the macro imports and compares all genotype calls from a batch of plates, read by both users. Any discrepancies are highlighted and the operator is prompted to resolve them manually.

- Transfer of all results in the results file. We have defined a sample as the concatenation of three pieces of information: sample_id, plate name and position of the well in the plate. This has been introduced in order to allow for the existence of samples duplicated on purpose.

- Duplication of a subset of samples, on the same plate or on different plates, is often used for quality control purposes. Knowing which samples have been duplicated for quality control, the macro looks for the results obtained for the first genotype and for the control genotype (identified in the TaqMan input and output files as "Qc_sample_id"), in order to compare them. The macro generates a quality control report file, where it flags all differences between samples and their controls, as well as their position in plates, to make the quality control checking easier.

### 3. Analysis

A macro called "**Count**" has also been created to make some basic statistical analysis of the results. For each SNP existing in the results file, it counts the frequency of each allele and of each genotype, and checks Hardy-Weinberg equilibrium, using a chi-square test.

Finally, we have added a macro "**Translate**", which converts the results from the four letters representing nucleotides into numbers. In further analysis, some pieces of software require numbers instead of letters for genotyping data, for instance Haploview [6] and/or TagSNPs [7,8]. The macro creates the starting point file for those applications. The user has still to arrange the polymorphisms according to their physical order and keep only the SNPs of interest depending e.g. on their frequencies and the blocks structure in the input file (TagSNPs software).

## Conclusion

We have created a set of user-friendly tools to make laboratory life easier and processing of genotyping data safer and quicker. The use of Microsoft Excel Visual Basic allows access to a wide range of users, working on PC or Mac computers.

This set of programs can be particularly helpful in laboratories where a full-fledged LIMS is not available. They complement (and can in the future be interfaced with) our database, which stores information on polymorphisms under study and their corresponding genotyping assays [9].

The macros we are presenting have been tailored to the instruments we have in our laboratory. However they can easily be modified in order to suit specific requirements, e.g. genotyping chemistries different from the 5' nuclease assay that can run on an Applied Biosystems Sequence Detection System, such as Amplifluor [10], or MGB Eclipse [11]. To this end, the source code of the macros can be freely consulted (see Additional file 1) and altered by using the Visual Basic Editor embedded in MS Excel.

## Availability and requirements

### Project name

TIMS, Macroshack.

### Project home page



### Operating system(s)

The software has been thoroughly tested in Excel 97 and 2000 under various versions of Windows (English versions), Excel 98 of MacOS 9 and Excel X of MacOS X (English versions).

### Programming language

Visual Basic. The source code of all programs is accessible by use of the Visual Basic Editor included in MS Excel.

### License

GNU General Public License.

### Any restrictions to use by non-academics?

None.

## Authors' contributions

FC has mainly conceived the tasks of the macros.

All the authors have contributed to writing the code.

SM and FC have prepared the first draft of the manuscript, and all co-authors contributed to the final draft.

## Supplementary Material

Additional File 1 Source code of software described in article. Visual Basic source code of macros described in the article. Each macro is composed of several modules or parts of Visual Basic code. Variables are defined by the key word "Dim" and comments are introduced after " ' ". Each piece of code is functional if embedded between "Sub NameOfTheSub" And "End Sub". Click here for file
