# Supplementary material for: T.I.M.S: TaqMan Information Management System, tools to organize data flow in a genotyping laboratory
Source: BMC Bioinformatics. 2005 Oct 12;6:246. doi: 10.1186/1471-2105-6-246 (PMC1266351; doi:10.1186/1471-2105-6-246)
Supplement: Additional File 1 — Source code of software described in article. Visual Basic source code of macros described in the article. Each macro is composed of several modules or parts of Visual Basic code. Variables are defined by the key word "Dim" and comments are introduced after " ' ". Each piece of code is functional if embedded between "Sub NameOfTheSub" And "End Sub". [file 1471-2105-6-246-S1.doc]

Code of programs described in

“T.I.M.S: TaqMan Information Management System, tools to organize data flow in a genotyping laboratory”

Each macro is composed of several modules, or pieces of code used by Visual Basic.

Variables are defined by the key word “Dim” and comments are introduced after “ ‘ ”.

Each piece of code is functional if embedded between “Sub NameoftheSub” And “End Sub”.

**1. “384To96.xls”**

- Module “Read Me”

Option Explicit

*' Purpose: This module contains a number of global variables and procedures.*

*' It also has the basic ReadMe procedure for displaying the Read Me worksheet*

Global blnMac As Boolean ' Global variable to indicate what system we are using

Global strMainWorkbook As String ' Name of the first workbook (usually 384 to96)

Sub ReadMe()

## 'Purpose: This procedure simply displayes the Read Me sheet

Sheets("Read Me").Activate

End Sub

Public Sub Initiate()

*'purpose : this procedure initiates the global variables for use in several of the main procedures.*

#If Mac Then 'determine our operating system type

blnMac = True

#Else

blnMac = False

#End If

strMainWorkbook = ActiveWorkbook.Name 'get the name of the initiating workbook

End Sub

- Module “Create96wellsplates”

Option Explicit

## 'Purpose: get the range of 96 wells from 384 wells plate

Sub Create96wellsplates()

Dim strFilename As String ' name of plate 384 processed

Dim intFile As Integer ' counter of batch

Dim strPlateName As String ' name of the plate file

Dim strPlatepath As String

Initiate 'Gets the OS type and the name of the main workbook.

Windows("384To96.xls").Activate

'ActiveWorkbook.Sheets("Data").Select

strPlatepath = ActiveWorkbook.Sheets("Data").Cells(1, 2) 'hidden sheet

#If Mac Then ' Use FileFind on Mac's, FileSearch on PC's

With Application.FileFind

.SearchPath = strPlatepath

#Else

With Application.FileSearch

.LookIn = strPlatepath

.FileType = msoFileTypeAllFiles

#End If

.Execute

For intFile = 1 To .FoundFiles.Count ' Work through each file in batch #1

strFilename = .FoundFiles(intFile)

Workbooks.Open strFilename ' Open the workbook

strPlateName = ActiveWorkbook.Name ' Get the name of the workbook

'treatement of the first plate

MakePlate 2, 2, 20

'treatement of the second plate

MakePlate 2, 3, 30

'treatement of the third plate

MakePlate 3, 2, 40

'treatement of the fourth plate

MakePlate 3, 3, 50

Workbooks(strPlateName).Save

Next

End With

Workbooks("384to96.xls").Activate

ActiveWorkbook.Close SaveChanges:=False

End Sub

Private Function MakePlate(ByVal vpt As Integer, ByVal hpt As Integer, ByVal strPlace As Integer) As Boolean

Dim LastCell As Integer ' row of the first empty cell

Dim x As Integer

Dim y As Integer

Dim i As Integer

Dim j As Integer

Dim c As Integer

MakePlate = False

LastCell = Columns(1).End(xlDown).Row + 1 'Get last empty cell

x = Rows(1).End(xlToRight).Column 'Get the last column, should be 25

y = Columns(2).End(xlDown).Row 'Get the last row , should be 17

Cells(19, 1) = "Plate96_1"

Cells(29, 1) = "Plate96_2"

Cells(39, 1) = "Plate96_3"

Cells(49, 1) = "Plate96_4"

' process line per line

For j = vpt To y

c = 1

'process column by column

For i = hpt To x

Cells(j, i).Select

Selection.Copy

Cells(strPlace, c).Select

ActiveSheet.Paste

c = c + 1 ' on avance sur la ligne

i = i + 1

Next i

strPlace = strPlace + 1

j = j + 1

Next j

Application.ScreenUpdating = False

MakePlate = True ' plate 96 wells processed

End Function

- Module “OpenplatefilesBatch”

Option Explicit

## 'Purpose : this macro opens the 384 plate maps

Sub OpenPlatesfiles()

Dim strPlateFile As String ' the folder containing plates maps

Dim strPlatepath As String ' the path of the folder

Dim ch As Integer ' counter used to reconstruct the path

Dim q As Integer ' counter used to reconstruct the path

Initiate 'Gets the OS type and name of the mina workbook

MsgBox "I will process all the files contained in the same folder" + vbCr + "If your folder contains other files, not corresponding to 384 map files, " + vbCr + "This will result in bugs!", vbExclamation, "Batch Files"

'Gets the name of the first files to be opened

strPlateFile = "False"

Do While strPlateFile = "False"

MsgBox "Please choose the folder containing your 384 plates", vbQuestion, "Batch Files Workbook"

strPlateFile = Application.GetOpenFilename

If strPlateFile = "False" Then 'Check for the cancel button

If MsgBox("No plates files has been selected. Do you want to open your plate file?", vbCritical + vbYesNo, "Open File ?") = vbNo Then

MsgBox "See you next time!", vbExclamation, "Bye Bye"

Exit Sub

End If

End If

Loop

MsgBox "Make sure your plate files are well formatted !", vbInformation, "Format File"

'Record the path of the file name in the Data worksheet

For ch = Len(strPlateFile) To 1 Step -1

If blnMac = True And Mid(strPlateFile, ch, 1) = ":" Then q = ch: GoTo 100

If blnMac = False And Mid(strPlateFile, ch, 1) = "\" Then q = ch: GoTo 100

Next ch

100 strPlatepath = Left(strPlateFile, q - 1)

'Sheets("Data").Select

ActiveWorkbook.Sheets("Data").Cells(1, 2) = strPlatepath

Sheets("Welcome!").Select

End Sub

**2. Macro “96to384”**

- Module “Read Me”

Option Explicit

Sub ReadMe()

*' Purpose: This module contains the basic ReadMe procedure for displaying the Read Me worksheet*

Dim ActiveWorkbook As Workbook

Sheets("Read Me").Activate

End Sub

- Module “Make 384plate_Batch”

## ‘Purpose: This module creates as many 384 wells plates as required

Sub Make384plate_batch()

Dim NumberofFiles As Byte

Dim InputFile As String

Dim PlateFile1 As String

Dim PlateFile2 As String

Dim PlateFile3 As String

Dim PlateFile4 As String

Dim Mac As Byte

Dim PC As Byte

Dim Rep As String

Dim F As Byte

'Determines whether the Wizard is run on Mac or PC

If Application.PathSeparator = ":" Then Mac = 1

If Application.PathSeparator = "\" Then PC = 1

BatchFile = False

'Asks for the file name of BatchFile

123 MsgBox "Please choose your Worklist File file: ", vbQuestion, "Batch File"

BatchFile = Application.GetOpenFilename

'Check for input file

Gestionnaire_error:

If BatchFile = False Then

Rep = MsgBox("No Worklist has been selected. Do you want to open your input file?", vbCritical + vbYesNo, "Open File ?")

Err.Number = 0

If Rep = vbYes Then

GoTo 123

Else: MsgBox " See you next time!", vbCritical, "Bye Bye"

Exit Sub

End If

End If

'Opens the BatchFile

Workbooks.Open BatchFile

MsgBox "Make sure your input file is well formatted !" + Chr(13), vbExclamation, "Worklist"

'Read data plates

Sheets("Sheet1").Select

i = 1

Range("A1").Select

Do While Not IsEmpty(Cells(i, 1))

Plate384Name = Cells(i, 1)

PlateFile1 = Cells(i + 1, 1)

PlateFile2 = Cells(i + 2, 1)

PlateFile3 = Cells(i + 3, 1)

PlateFile4 = Cells(i + 4, 1)

RearrMode = Cells(i + 5, 1)

'Creates 384-well plate file

Workbooks.Add

'Saves 384-well plate file

ActiveWorkbook.SaveAs Filename:=Plate384Name + ".xls", FileFormat:=xlNormal, Password _

:="", WriteResPassword:="", ReadOnlyRecommended:=False, CreateBackup:= _

False

'Saves the name of the workbook

File384Name = ActiveWorkbook.Name

'Renames worksheet 1 as the name of the 384-well plate

Sheets("Sheet1").Select

Sheets("Sheet1").Name = Plate384Name

'Formats the 384-well plate file

Cells(1, 1) = Plate384Name

'Writes column numbers

For xx = 1 To 24

Cells(1, 1 + xx) = xx

Next xx

'Writes row letters

Cells(2, 1) = "A"

Cells(3, 1) = "B"

Cells(4, 1) = "C"

Cells(5, 1) = "D"

Cells(6, 1) = "E"

Cells(7, 1) = "F"

Cells(8, 1) = "G"

Cells(9, 1) = "H"

Cells(10, 1) = "I"

Cells(11, 1) = "J"

Cells(12, 1) = "K"

Cells(13, 1) = "L"

Cells(14, 1) = "M"

Cells(15, 1) = "N"

Cells(16, 1) = "O"

Cells(17, 1) = "P"

'Draws borders

Range("B2:Y17").Select

Selection.Borders(xlEdgeLeft).LineStyle = xlContinuous

Selection.Borders(xlEdgeTop).LineStyle = xlContinuous

Selection.Borders(xlEdgeBottom).LineStyle = xlContinuous

Selection.Borders(xlEdgeRight).LineStyle = xlContinuous

Selection.Borders(xlInsideVertical).LineStyle = xlContinuous

Selection.Borders(xlInsideHorizontal).LineStyle = xlContinuous

Range("A1").Select

'Treatement of the first plate

Workbooks.Open PlateFile1

PlateFileName1 = ActiveWorkbook.Name

'Copies 96-well plate_1 into 384-well plate file

Windows(PlateFileName1).Activate

Application.CutCopyMode = False

Range("A1:M9").Copy Destination:=Workbooks(File384Name).Sheets(Plate384Name).Range("A19")

Windows(PlateFileName1).Activate

ActiveWorkbook.Close

'Treatement of the second plate

Workbooks.Open PlateFile2

PlateFileName2 = ActiveWorkbook.Name

'Copies 96-well plate_2 into 384-well plate file

Windows(PlateFileName2).Activate

Application.CutCopyMode = False

Range("A1:M9").Copy Destination:=Workbooks(File384Name).Sheets(Plate384Name).Range("A29")

Windows(PlateFileName2).Activate

ActiveWorkbook.Close

'Treatement of the third plate

Workbooks.Open PlateFile3

PlateFileName3 = ActiveWorkbook.Name

'Copies 96-well plates_3 into 384-well plate file

Windows(PlateFileName3).Activate

Application.CutCopyMode = False

Range("A1:M9").Copy Destination:=Workbooks(File384Name).Sheets(Plate384Name).Range("A39")

Windows(PlateFileName3).Activate

ActiveWorkbook.Close

'Treatement of the fourth plate

Workbooks.Open PlateFile4

PlateFileName4 = ActiveWorkbook.Name

'Copies 96-well plates_4 into 384-well plate file

Windows(PlateFileName4).Activate

Application.CutCopyMode = False

Range("A1:M9").Copy Destination:=Workbooks(File384Name).Sheets(Plate384Name).Range("A49")

Windows(PlateFileName4).Activate

ActiveWorkbook.Close

'Minimizes 384-well plate file window

Windows(File384Name).Activate

If Mac = 1 Then

With ActiveWindow

.Width = 100

.Height = 100

End With

End If

If PC = 1 Then

ActiveWindow.WindowState = xlNormal

With ActiveWindow

.Width = 100

.Height = 100

End With

End If

'Starts copying of plate contents according to rearrangement mode

If RearrMode = "Z" Then GoTo 200

If RearrMode = "N" Then GoTo 300

200

'Copies the content of 96-well plate 1 into the 384-well plate scheme (Z-mode)

For xx = 1 To 12

For yy = 1 To 8

Range(Cells(yy + 19, xx + 1), Cells(yy + 19, xx + 1)).Select

Selection.Copy

Range(Cells(yy * 2, xx * 2), Cells(yy * 2, xx * 2)).Select

ActiveSheet.Paste

Next yy

Next xx

'Copies the content of 96-well plate 2 into the 384-well plate scheme (Z-mode)

For xx = 1 To 12

For yy = 1 To 8

Range(Cells(yy + 29, xx + 1), Cells(yy + 29, xx + 1)).Select

Selection.Copy

Range(Cells(yy * 2, xx * 2 + 1), Cells(yy * 2, xx * 2 + 1)).Select

ActiveSheet.Paste

Next yy

Next xx

'Copies the content of 96-well plate 3 into the 384-well plate scheme (Z-mode)

For xx = 1 To 12

For yy = 1 To 8

Range(Cells(yy + 39, xx + 1), Cells(yy + 39, xx + 1)).Select

Selection.Copy

Range(Cells(yy * 2 + 1, xx * 2), Cells(yy * 2 + 1, xx * 2)).Select

ActiveSheet.Paste

Next yy

Next xx

'Copies the content of 96-well plate 4 into the 384-well plate scheme (Z-mode)

For xx = 1 To 12

For yy = 1 To 8

Range(Cells(yy + 49, xx + 1), Cells(yy + 49, xx + 1)).Select

Selection.Copy

Range(Cells(yy * 2 + 1, xx * 2 + 1), Cells(yy * 2 + 1, xx * 2 + 1)).Select

ActiveSheet.Paste

Next yy

Next xx

GoTo 400

300

'Copies the content of 96-well plates into the 384-well plate scheme (inverted N-mode)

'Copies the content of 96-well plate 1 into the 384-well plate scheme (inverted-mode)

For xx = 1 To 12

For yy = 1 To 8

Range(Cells(yy + 19, xx + 1), Cells(yy + 19, xx + 1)).Select

Selection.Copy

Range(Cells(yy * 2, xx * 2), Cells(yy * 2, xx * 2)).Select

ActiveSheet.Paste

Next yy

Next xx

'Copies the content of 96-well plate 2 into the 384-well plate scheme (inverted-mode)

For xx = 1 To 12

For yy = 1 To 8

Range(Cells(yy + 29, xx + 1), Cells(yy + 29, xx + 1)).Select

Selection.Copy

Range(Cells(yy * 2 + 1, xx * 2), Cells(yy * 2 + 1, xx * 2)).Select

ActiveSheet.Paste

Next yy

Next xx

'Copies the content of 96-well plate 3 into the 384-well plate scheme (inverted-mode)

For xx = 1 To 12

For yy = 1 To 8

Range(Cells(yy + 39, xx + 1), Cells(yy + 39, xx + 1)).Select

Selection.Copy

Range(Cells(yy * 2, xx * 2 + 1), Cells(yy * 2, xx * 2 + 1)).Select

ActiveSheet.Paste

Next yy

Next xx

'Copies the content of 96-well plate 4 into the 384-well plate scheme (inverted-mode)

For xx = 1 To 12

For yy = 1 To 8

Range(Cells(yy + 49, xx + 1), Cells(yy + 49, xx + 1)).Select

Selection.Copy

Range(Cells(yy * 2 + 1, xx * 2 + 1), Cells(yy * 2 + 1, xx * 2 + 1)).Select

ActiveSheet.Paste

Next yy

Next xx

400

'Deletes the 4 96-well plates

Rows("19:57").Select

Selection.Delete Shift:=xlUp

Range("B2").Select

'Copies the rearrangement mode

Cells(20, 1) = "Rearrangement Mode:"

If RearrMode = "N" Then

Cells(20, 2) = Left(PlateFileName1, Len(PlateFileName1) - 4)

Cells(21, 2) = Left(PlateFileName2, Len(PlateFileName2) - 4)

Cells(20, 3) = Left(PlateFileName3, Len(PlateFileName3) - 4)

Cells(21, 3) = Left(PlateFileName4, Len(PlateFileName4) - 4)

Else

Cells(20, 2) = Left(PlateFileName1, Len(PlateFileName1) - 4)

Cells(20, 3) = Left(PlateFileName2, Len(PlateFileName2) - 4)

Cells(21, 2) = Left(PlateFileName3, Len(PlateFileName3) - 4)

Cells(21, 3) = Left(PlateFileName4, Len(PlateFileName4) - 4)

End If

'Restores 384-well file window to its original size

Windows(File384Name).Activate

ActiveWindow.WindowState = xlMaximized

'Completes, saves and close the 384-well file

Cells.Select

Cells.EntireColumn.AutoFit

Range("B2").Select

ActiveWindow.FreezePanes = True

Range("A1").Select

ActiveWorkbook.Save

'Offers the possiblity to make TaqMan Setup Table

If (MsgBox("You have, now, the possiblity to create the TaqMan input files for the newly created 384-well plate. " + Chr(13) + Chr(13) + "These files are useful if you plan to genotype the samples with the TaqMan robot." + Chr(13) + Chr(13) + "Do you want to create the TaqMan Input Files?", vbQuestion + vbYesNo, "TaqMan Input Files?") = vbNo) Then

ActiveWorkbook.Close SaveChanges = True

GoTo 100 ' Goes on with the next 384- wells plate

Else

'Formats the TaqMan setup table sheet if the user wants to

'Creates SetupTableFile

Workbooks.Add

SetupTableFileName = ActiveWorkbook.Name

Sheets("Sheet1").Activate

Cells(1, 1) = "*** SDS Setup File Version"

Cells(1, 2) = 3

Cells(2, 1) = "*** Output Plate Size"

Cells(2, 2) = 384

Cells(3, 1) = "*** Output Plate ID"

Cells(3, 2) = Plate384Name

Cells(4, 1) = "*** Number of Detectors"

Cells(4, 2) = 0

Cells(5, 1) = "Detector"

Cells(5, 2) = "Reporter"

Cells(5, 3) = "Quencher"

Cells(5, 4) = "Description"

Cells(5, 5) = "Comments"

Cells(6, 1) = "Well"

Cells(6, 2) = "Sample Name"

Cells(6, 3) = "Detector"

Cells(6, 4) = "Task"

Cells(6, 5) = "Quantity"

For yy = 1 To 384

Cells(6 + yy, 1) = yy

Next yy

j = 0

'Copies sample names from the plate file to the TaqMan setup table

For yy = 1 To 16

Windows(File384Name).Activate

Range(Cells(yy + 1, 2), Cells(yy + 1, 25)).Select

Application.CutCopyMode = False

Selection.Copy

Windows(SetupTableFileName).Activate

Range(Cells(j + 7, 2), Cells(j + 7, 2)).Select

Selection.PasteSpecial Paste:=xlValues, Operation:=xlNone, SkipBlanks:=False, Transpose:=True

j = j + 24

Next yy

'Writes "NTC" where the sample name is missing

Windows(SetupTableFileName).Activate

For yy = 7 To 390

If Cells(yy, 2) = "" Then Cells(yy, 2) = "NTC"

Next yy

'Saves TaqMan setup table file

ActiveWorkbook.SaveAs Filename:=Plate384Name + ".txt", FileFormat:=xlText, Password:="", WriteResPassword:="", ReadOnlyRecommended:=False, CreateBackup:=False

ActiveWorkbook.Close SaveChanges = True

Windows(File384Name).Activate

ActiveWorkbook.Close

End If

'Opens the BatchFile

100 Workbooks.Open BatchFile

'Read data plates

Sheets("Sheet1").Select

i = i + 6

Loop

MsgBox "Your 384-well plates and TaqMan Input Files (if you have chosen to make them) have been created with the worklist names " + Chr(13) + Chr(13) + "They are located in the same folder as the one containing the original plates.", vbInformation, "Execution Completed !"

ActiveWorkbook.Close SaveChanges = False

End Sub

**3.Macro “Plate2TaqMan”**

- Module “Read Me”

Sub ReadMe()

*' Purpose: This module contains the basic ReadMe procedure for displaying the Read Me worksheet*

'Dim ActiveWorkbook As Workbook

Sheets("Read Me").Select

End Sub

- Module “OpenBatchFile”

Sub OpenBatchFile()

'

'Determines whether the Wizard is run on Mac or PC

If Application.PathSeparator = ":" Then Mac = 1

If Application.PathSeparator = "\" Then PC = 1

BatchFile = False

'Asks for the plate file name

MsgBox "Please choose your batch file with the plate maps ", vbQuestion, "Batch File?"

150 BatchFile = Application.GetOpenFilename

'Gestion of Cancel

If BatchFile = "False" Then 'Check for the cancel button

If MsgBox("None plate file has been selected. Do you want to open your plates file?", vbCritical + vbYesNo, "OpenFile?") = vbNo Then

MsgBox "See next time", vbExclamation, "Bye Bye"

Exit Sub

Else: GoTo 150

End If

End If

'Opens the plate file

Workbooks.Open BatchFile

' Record the path in the data sheet

For ch = Len(BatchFile) To 1 Step -1

If Mac = 1 And Mid(BatchFile, ch, 1) = ":" Then q = ch: GoTo 100

If PC = 1 And Mid(BatchFile, ch, 1) = "\" Then q = ch: GoTo 100

Next ch

100 PlateFilePath = Left(BatchFile, q - 1)

Windows("Plate2TaqMan.xls").Activate

' Sheets("Data").Select

ActiveWorkbook.Sheets("Data").Cells(1, 1) = PlateFilePath

Sheets("Welcome!").Select

MsgBox "Make sure the plate file is in the correct format!", vbExclamation, "Be Careful"

End Sub

- Module “MakeTaqManTable”

Public RearrMode

Sub MakeTaqManTable()

'

'Determines whether the Wizard is run on Mac or PC

If Application.PathSeparator = ":" Then Mac = 1

If Application.PathSeparator = "\" Then PC = 1

'Reads plate file name

' Sheets("Data").Select

PlateFilePath = ActiveWorkbook.Sheets("Data").Cells(1, 1)

Sheets("Welcome!").Select

'Batch process

'Use FileFind on Mac, File Search on PC

#If Mac Then

With Application.FileFind

.SearchPath = PlateFilePath

#Else

With Application.FileSearch

.LookIn = PlateFilePath

.FileType = msoFileTypeAllFiles

#End If

.Execute

For F = 1 To .FoundFiles.Count

strFilename = .FoundFiles(F) 'whole name ( path +file)

Workbooks.Open strFilename

PlateFileName = ActiveWorkbook.Name 'shorter name (file)

'Checks whether plate is 96 or 384 wells

Windows(PlateFileName).Activate

If Cells(10, 1) = "" Then Wells = 96: Colonne = 12: Righe = 8

If Cells(10, 1) = "I" Then Wells = 384: Colonne = 24: Righe = 16

'Formats the TaqMan setup table sheet

Workbooks.Add

SetupTableFileName = ActiveWorkbook.Name

'SetupTableFileName = Left(PlateFileName, Len(PlateFileName) - 4) '+ ".txt"

Cells(1, 1) = "*** SDS Setup File Version"

Cells(1, 2) = 3

Cells(2, 1) = "*** Output Plate Size"

Cells(2, 2) = Wells

Cells(3, 1) = "*** Output Plate ID"

Cells(3, 2) = (Left(PlateFileName, Len(PlateFileName) - 4))

Cells(4, 1) = "*** Number of Detectors"

Cells(4, 2) = 0

Cells(5, 1) = "Detector"

Cells(5, 2) = "Reporter"

Cells(5, 3) = "Quencher"

Cells(5, 4) = "Description"

Cells(5, 5) = "Comments"

Cells(6, 1) = "Well"

Cells(6, 2) = "Sample Name"

Cells(6, 3) = "Detector"

Cells(6, 4) = "Task"

Cells(6, 5) = "Quantity"

For yy = 1 To Wells

Cells(6 + yy, 1) = yy

Next yy

xx = 0

'Copies sample names from the plate file to the TaqMan setup table

For yy = 1 To Righe

Windows(PlateFileName).Activate

Range(Cells(yy + 1, 2), Cells(yy + 1, Colonne + 1)).Select

Application.CutCopyMode = False

Selection.Copy

Windows(SetupTableFileName).Activate

Range(Cells(xx + 7, 2), Cells(xx + 7, 2)).Select

Selection.PasteSpecial Paste:=xlValues, Operation:=xlNone, SkipBlanks:=False, Transpose:=True

xx = xx + Colonne

Next yy

'Writes "NTC" where the sample name is missing

Windows(SetupTableFileName).Activate

For yy = 7 To Wells + 6

If Cells(yy, 2) = "" Then Cells(yy, 2) = "NTC"

Next yy

'Saves TaqMan setup table file

Windows(SetupTableFileName).Activate

'Saves the new workbook as a text file, independently from the Excel workbook

ActiveWorkbook.SaveAs Filename:=Left(PlateFileName, Len(PlateFileName) - 4) + ".txt", FileFormat:=xlText

'Saves the new workbook as a text file, independently from the Excel workbook

ActiveWorkbook.Close SaveChanges:=False

Windows(PlateFileName).Activate

ActiveWorkbook.Close

Next F

End With

End Sub

**4.Macro “ResultsFileBuilder”**

- Module “ReadMe”

Sub ReadMe()

' Sheets("Read me").Select

Range("A6").Select

End Sub

- Module “OpenPlateBatch”

Sub OpenPlateBatch()

## ‘Purpose: this module open the folder containing the plates files to process

Dim PlateFile As String

'

'Determines whether the Wizard is run on Mac or PC

If Application.PathSeparator = ":" Then Mac = 1

If Application.PathSeparator = "\" Then PC = 1

MsgBox "I will process all and only the files contained in the same folder," + Chr(13) + "and also all the ones contained in all subfolders (Mac only)!" + Chr(13) + Chr(13) + "If the folder contains other files, not corresponding to plate files," + Chr(13) + "this will result in bugs!", vbExclamation, "Plates Files?"

'Asks for the file name

PlateFile = False

MsgBox "Please choose the first file with the plate map", vbQuestion, "Plate Map"

150 PlateFile = Application.GetOpenFilename

' if cancel button

If PlateFile = "False" Then ' Check for the cancel button

If MsgBox("No plates files has been selectd. Do you want to open your plate files ?", vbCritical + vbYesNo, "Open File") = vbNo Then

MsgBox "See you next time !", vbExclamation, "Bye Bye"

Exit Sub

Else: GoTo 150

End If

End If

'Reconstructs the path

For ch = Len(PlateFile) To 1 Step -1

If Mac = 1 And Mid(PlateFile, ch, 1) = ":" Then q = ch: GoTo 100

If PC = 1 And Mid(PlateFile, ch, 1) = "\" Then q = ch: GoTo 100

Next ch

100 PlateFilePath = Left(PlateFile, q - 1)

Windows("ResultsFileBuilder.xls").Activate

'Sheets("Data").Select

ActiveWorkbook.Sheets("Data").Cells(5, 2) = PlateFilePath

Sheets("Welcome!").Select

End Sub

- Module “DoTransferBatch”

Option Compare Text

‘Purpose: creates the results files, transferring the samples names in a new file

Sub DoTransferBatch()

'

'Determines whether the Wizard is run on Mac or PC

If Application.PathSeparator = ":" Then Mac = 1

If Application.PathSeparator = "\" Then PC = 1

'If the PC macro has been started by mistake, it warns and stops the macro

'If Mac = 1 Then MsgBox "You should use the Make results file (Mac) function.": GoTo 99999

'Creates data transfer matrixes

Dim SampleNameTransfer()

'Reads file names

'Sheets("Data").Select

PlateFilePath = ActiveWorkbook.Sheets("Data").Cells(5, 2)

Sheets("Welcome!").Select

'Asks for the name of the results file

ResFileGu = InputBox("Enter the name to give to the results file.")

'Creates results file

Workbooks.Add

'Asks where to save the results file

MsgBox "Please choose where you want to save your results file"

25 ResFile = Application.GetSaveAsFilename(initialFilename:=ResFileGu + ".xls")

If Right(ResFile, 4) <> ".xls" Then

If Mac = 1 Then ResFile = ResFile + ".xls"

If PC = 1 Then ResFile = ResFile + "xls"

End If

For ch = Len(ResFile) To 1 Step -1

If Mac = 1 And Mid(ResFile, ch, 1) = ":" Then q = ch: GoTo 10

If PC = 1 And Mid(ResFile, ch, 1) = "\" Then q = ch: GoTo 10

Next ch

10 ResFileName = Right(ResFile, Len(ResFile) - q)

ResFilePath = Left(ResFile, q - 1)

If ResFilePath = PlateFilePath Then

MsgBox "Please, choose another folder", vbExclamation, "Another Folder?"

GoTo 25

Else: GoTo 22

End If

'Saves results file

22 ActiveWorkbook.SaveAs Filename:=ResFile, FileFormat:=xlNormal, Password _

:="", WriteResPassword:="", ReadOnlyRecommended:=False, CreateBackup:= _

False

'Renames worksheet 1

Sheets("Sheet1").Select

Sheets("Sheet1").Name = ResFileGu + " results"

'Formats the results file

Cells.Select

With Selection

.HorizontalAlignment = xlCenter

.VerticalAlignment = xlCenter

End With

Cells(1, 1) = ResFileGu

Cells(2, 1) = ResFileGu

Cells(1, 2) = "Plate"

Cells(1, 3) = "Row"

Cells(1, 4) = "Column"

Cells(2, 2) = "-"

Cells(2, 3) = "-"

Cells(2, 4) = "-"

Range("B3").Select

ActiveWindow.FreezePanes = True

yr = 3

'Adds progress bar

Windows("ResultsFileBuilder.xls").Activate

Sheets("Progress").Select

ActiveSheet.Shapes.AddShape(msoShapeRectangle, 198, 28, 204, 14).Select

Selection.ShapeRange.Fill.Visible = msoFalse

'Batch process

'Use FileFind on Mac, File Search on PC

#If Mac Then

With Application.FileFind

.SearchPath = PlateFilePath

#Else

With Application.FileSearch

.LookIn = PlateFilePath

.FileType = msoFileTypeAllFiles

#End If

.Execute

20 For F = 1 To .FoundFiles.Count

'''''BEGINS ADDING PLATE FILES (PlateFile)

'Opens plate file

PlateFile = .FoundFiles(F)

Workbooks.Open PlateFile

PlateFileName = Right(PlateFile, Len(PlateFile) - Len(PlateFilePath) - 1)

'Reads plate name

PlateName = Cells(1, 1)

'Determines whether it is 96 or 384 and dimensions transfer matrix

If Cells(1, 14) = 13 Then

PlateType = 384

Colonne = 24

Righe = 16

Else

PlateType = 96

Colonne = 12

Righe = 8

End If

ProgressIncrement = 200 / .FoundFiles.Count

ReDim SampleNameTransfer(Righe + 1, Colonne + 1)

'Writes column numbers into transfer matrix

For xx = 2 To Colonne + 1

SampleNameTransfer(1, xx) = xx - 1

Next xx

'Writes row letters into transfer matrix

SampleNameTransfer(2, 1) = "A"

SampleNameTransfer(3, 1) = "B"

SampleNameTransfer(4, 1) = "C"

SampleNameTransfer(5, 1) = "D"

SampleNameTransfer(6, 1) = "E"

SampleNameTransfer(7, 1) = "F"

SampleNameTransfer(8, 1) = "G"

SampleNameTransfer(9, 1) = "H"

If PlateType = 96 Then GoTo 29

SampleNameTransfer(10, 1) = "I"

SampleNameTransfer(11, 1) = "J"

SampleNameTransfer(12, 1) = "K"

SampleNameTransfer(13, 1) = "L"

SampleNameTransfer(14, 1) = "M"

SampleNameTransfer(15, 1) = "N"

SampleNameTransfer(16, 1) = "O"

SampleNameTransfer(17, 1) = "P"

'Copies data into transfer matrix

29 For xx = 2 To Colonne + 1

For yy = 2 To Righe + 1

SampleNameTransfer(yy, xx) = Cells(yy, xx)

Next yy

Next xx

'Closes plate file

ActiveWorkbook.Close SaveChanges:=False

'Goes to results file

Windows(ResFileName).Activate

'Pastes data into results file

For xx = 2 To Colonne + 1

For yy = 2 To Righe + 1

If SampleNameTransfer(yy, xx) = "" Then GoTo 35

Cells(yr, 1) = SampleNameTransfer(yy, xx)

Cells(yr, 2) = PlateName

Cells(yr, 3) = SampleNameTransfer(yy, 1)

Cells(yr, 4) = SampleNameTransfer(1, xx)

yr = yr + 1

35 Next yy

Next xx

'Updates progress bar

Windows("ResultsFileBuilder.xls").Activate

Sheets("Progress").Select

'...erases the old one...

For Each s In Sheets("Progress").Shapes

s.Delete

Next

'...redraws the frame...

ActiveSheet.Shapes.AddShape(msoShapeRectangle, 198, 28, 204, 14).Select

Selection.ShapeRange.Fill.Visible = msoFalse

'...calculates the length of the new progress bar and draws it

Progress = Progress + ProgressIncrement

ActiveSheet.Shapes.AddShape(msoShapeRectangle, 200, 30, Progress, 10).Select

Selection.ShapeRange.Fill.ForeColor.RGB = RGB(255, 51, 153)

Selection.ShapeRange.Fill.BackColor.RGB = RGB(51, 102, 255)

Selection.ShapeRange.Fill.PresetGradient msoGradientVertical, 1, msoGradientRainbowII

Next F

End With

'Does final formatting of the results file

Windows(ResFileName).Activate

Cells.Select

Cells.EntireColumn.AutoFit

ActiveWorkbook.Save

Range(Cells(yr, 2), Cells(yr, 2)).Select

10000

'Erases the progress bar

Windows("ResultsFileBuilder.xls").Activate

Sheets("Progress").Select

For Each s In Worksheets("Progress").Shapes

s.Delete

Next

Sheets("Welcome!").Select

99999

Windows("ResultsFileBuilder.xls").Activate

ActiveWorkbook.Close SaveChanges:=False

End Sub

**5. Macro “Translate”**

- Module “Read Me”

Option Explicit

Sub ReadMe()

Dim ActiveWorkbook As Workbook

Sheets("Read Me").Activate

End Sub

- Module “Translate”

Option Compare Text

Sub Translation()

*' Purpose: This procedure translates allele values from the Results workbook.*

Dim intAll(1 To 2) As Integer ' Allele values

Dim shtTranslation As Worksheet ' Pointer to the Translation worksheet in the results workbook

Dim intRow As Integer ' used to loop through each row in the results worksheet

Dim intMarker As Integer ' Used to loop through each marker in the results worksheet

Dim ResultfileName As String

'Inform the user that he 's going to open his resultfile

100 MsgBox "Please choose your results file", vbQuestion, "Results Workbook"

On Error GoTo Gestionnnaire_error

‘open explorer

ResultFile = Application.GetOpenFilename

' check for input file

Gestionnnaire_error:

If ResultFile = False Then

Rep = MsgBox("None results file has been selected. Do you want to open your resultsfile ?", vbCritical + vbYesNo, "Open file ?")

'Resume

' Err.Number = 0

If Rep = vbYes Then

GoTo 100

Else: MsgBox "See you next time!", vbCritical, "Bye Bye"

Exit Sub

End If

End If

' Opens effectively the results file

Workbooks.Open ResultFile

'Gets the name of the workbook

ResultfileName = ActiveWorkbook.Name

MsgBox "Make sure " + ResultfileName + " is well formatted !", vbInformation

'OpenResultFile.OpenResultFile (False) ' Open the results file (not updating the main worksheet)

Application.ScreenUpdating = False ' Stop updating (prevents screen flicker)

ActiveSheet.UsedRange.Copy ' Copy the data from the first worksheet

Set shtTranslation = Worksheets.Add ' Add a new worksheet to the results workbook

shtTranslation.Name = "Translation" ' Rename it, paste the data and select it

shtTranslation.Paste

shtTranslation.Select

intMarker = 5 ' markers start at column 5

Do While Not IsEmpty(Cells(1, intMarker)) ' Process all markers

If Cells(2, intMarker) <> "-" Then ' Skip - entries

intRow = 3 ' Allele values start at row 3

Do While Not IsEmpty(Cells(intRow, 1)) ' Process all results

intAll(1) = 0

intAll(2) = 0

If Cells(intRow, intMarker) <> "" Then ' Skip blank entries

' Convert the allele values from ACGT to 1234 (- = 0)

intAll(1) = InStr("ACGT", Left$(Cells(intRow, intMarker), 1))

intAll(2) = InStr("ACGT", Right$(Cells(intRow, intMarker), 1))

' Enter the converted values

Cells(intRow, intMarker) = intAll(1) & " " & intAll(2)

End If

intRow = intRow + 1

Loop

intMarker = intMarker + 1

End If

Loop

Set shtTranslation = Nothing ' Get rid of the pointer

Application.ScreenUpdating = True

MsgBox "Translation completed"

End Sub

**6. Macro “TaqMan2Results”**

# Module “Read Me”

Option Explicit

*' Purpose: This module contains a number of global variables and procedures.*

*' It also has the basic ReadMe procedure for displaying the Read Me worksheet*

Global blnMac As Boolean ' Global variable to indicate what system we are using

Global strMainWorkbook As String ' Name of the first workbook (usually TaqMan2Results.xls)

Sub ReadMe()

' Purpose: This procedure simply displayes the Read Me sheet

Sheets("Read Me").Activate ' Display the Read me sheet

End Sub

Public Sub Initiate()

*' Purpose: This procedure initiates the global variables for use in several of the main procedures.*

#If Mac Then ' Determine our operating system type

blnMac = True

#Else

blnMac = False

#End If

strMainWorkbook = ActiveWorkbook.Name ' Get the name of the initiating workbook

End Sub

# Module “OpenResultFile”

Option Explicit

*' Purpose: This macro opens the results file.*

Public Sub OpenResultFile(Optional ByVal blnUpdateMain As Boolean = True)

' Purpose: This procedure opens the results worksheet.

' It is called from the main worksheet or from the translation function.

' If called from the main worksheet it will record it's name into the Data worksheet

Dim strFilename As String ' The path of the results file

Dim strResultFileName As String ' The name of the results file

Initiate ' Get the OS type and name of the main workbook

' Get the name of the results file

strFilename = "False"

Do While strFilename = "False"

MsgBox "Please choose your results file", vbQuestion, "Results Workbook"

strFilename = Application.GetOpenFilename

If strFilename = "False" Then ' Check for the Cancel key

If MsgBox("No results file has been selected. Do you want to open your results file ?", _

vbCritical + vbYesNo, "Open file ?") = vbNo Then

MsgBox "See you next time!", vbCritical, "Bye Bye"

Exit Sub

End If

End If

Loop

Workbooks.Open strFilename ' Open the results file

strResultFileName = ActiveWorkbook.Name

MsgBox "Make sure " + strResultFileName + " is well formatted !", vbInformation

If blnUpdateMain Then

Windows(strMainWorkbook).Activate ' And record it's name in the data sheet

Worksheets("Data").Cells(4, 2) = strResultFileName

Sheets("Welcome !").Select ' Display the welcome screen again

End If

End Sub

# Module “OpenPlateFileBatch1”

Option Explicit

*' Purpose: These macros open the two plate files.*

Sub OpenPlateFileBatch1()

OpenFile 1 ' Get the first plate file

End Sub

Sub OpenPlateFileBatch2()

OpenFile 2 ' Get the second plate file

End Sub

Sub OpenFile(ByVal intFileNum As Integer)

Dim strFilename As String ' The name of the plate file to import

Dim strFilePath As String ' The path of the plate file to import

Dim intLast As Integer ' Row index of the first empty cell in column 4

Dim ch As Integer ' counter used to replace InsStrRev 'cos doesn't work on mac...

Dim q As Integer ' counter used to replace InsStrRev 'cos doesn't work on mac...

Initiate ' Get the OS type and name of the main workbook

MsgBox "I will process all and only the files contained in the same folder," + _

vbCr + "and also all the ones contained in all subfolders (Mac only)!" + _

vbCr + vbCr + "If the folder contains other files, not corresponding to TaqMan files," + _

vbCr + "this will result in bugs!", vbExclamation, "BatchFiles"

' Get the name of one of the files to be loaded

strFilename = "False"

Do While strFilename = "False"

MsgBox "Please choose the " & Choose(intFileNum, "first", "second") & " batch of your plate results", _

vbQuestion, "Batch " & intFileNum & " Workbook"

strFilename = Application.GetOpenFilename

If strFilename = "False" Then ' Check for the Cancel button

If MsgBox("None results file has been selected. Do you want to open your TaqMan file ?", _

vbCritical + vbYesNo, "Open file ?") = vbNo Then

MsgBox "See you next time!", vbCritical, "Bye Bye"

Exit Sub

End If

End If

Loop

MsgBox "Make sure your plate files are well formatted !", vbInformation, "Format File ?"

' Record the path of the filename in the Data worksheet

'strFilePath = Left$(strFilename, InStrRev(strFilename, IIf(blnMac, ":", "\")) - 1)

For ch = Len(strFilename) To 1 Step -1

If blnMac = True And Mid(strFilename, ch, 1) = ":" Then q = ch: GoTo 100

If blnMac = False And Mid(strFilename, ch, 1) = "\" Then q = ch: GoTo 100

Next ch

100 strFilePath = Left(strFilename, q - 1)

Sheets("Data").Select

If intFileNum = 1 Then Range("B2: B5").Delete ' Clear the results if we selected the first file

Cells(1 + intFileNum, 2) = strFilePath ' Record the file path

' Also store it in the Run Report worksheet.

' **** This seems strange as the second plate name will overwrite the first and they will both

' **** be overwritten during the PasteData phase

'Sheets("Run Report").Select

'intLast = Cells(1, 4).End(xlDown).Row + 1 ' Find the first empty cell

'Cells(intLast, 1) = strFilePath ' And record the path

Sheets("Welcome !").Select ' Go back to the Welcome worksheet

End Sub

# Module “Paste Data”

Option Explicit

Option Compare Text

*' Purpose: This module contains procedures for transferring data from the two plate files*

*' into the Temp worksheet.*

Dim intPlate As Integer ' Number of wells on each plate

Sub PasteData()

Dim strPlate2FileName As String ' Name of the second plate file

Dim strResultFileName As String ' Name of the results file

Dim IntLoop As Integer ' Generic looping variable

Dim StrGeno(1 To 2) As String ' Results

Dim intPlate As Integer ' Number of samples on each plate

Dim strRunReportFile As String ' Name of the report file to save to (includes full path)

Dim strRunReportFileName As String ' Name of the report spreadsheet

Dim blnDbleReading As Boolean ' Double reading is optionnal

Dim lngDiscr As Long ' number of discrepencies found

Dim lngMiss As Long ' number of missing found

Initiate ' Get the OS type and name of the main workbook

Application.ScreenUpdating = False ' Stop updating (prevents screen flicker)

Sheets("Data").Select ' Get the plate and results file names

strPlate2FileName = Cells(3, 2)

If Not IsEmpty(Cells(3, 2)) Then ' Double reading is optionnal

blnDbleReading = True

End If

strResultFileName = Cells(4, 2)

' Import the two files

If Not AnalyseFile(Cells(2, 2), 1, strResultFileName) Then Exit Sub

If blnDbleReading = "True" Then

If Not AnalyseFile(strPlate2FileName, 2, strResultFileName) Then Exit Sub

End If

'''''''''''''''''''''''''''Compare both readings '''''''''''''''''''''''''

Sheets("Temp ").Select

IntLoop = 3

lngDiscr = 0

lngMiss = 0

If blnDbleReading = "True" Then

Do While Cells(IntLoop, 1) <> "" ' Process all the rows in the temp worksheet

StrGeno(1) = Cells(IntLoop, 5)

StrGeno(2) = Cells(IntLoop, 6)

If StrGeno(1) = StrGeno(2) Then Cells(IntLoop, 7) = StrGeno(1)

If StrGeno(1) <> StrGeno(2) Then

Cells(IntLoop, 7) = "First Reading= " + StrGeno(1) + " + " + "Second Reading= " + StrGeno(2)

lngDiscr = lngDiscr + 1 'counts the number of discrepencies found

End If

If StrGeno(1) = "" Then Cells(IntLoop, 7) = "First Reading is missing" + " + " + "Second Reading= " + StrGeno(2)

If StrGeno(2) = "" Then Cells(IntLoop, 7) = "First Reading= " + StrGeno(1) + " + " + "Second Reading is missing "

If Cells(IntLoop, 7) = "-" Then lngMiss = lngMiss + 1

Cells(IntLoop, 3) = GetCell(Cells(IntLoop, 3), IIf(intPlate = 96, 12, 24)) ' Translate the cells from values to grid references

Cells(IntLoop, 8) = ""

IntLoop = IntLoop + 1

Loop

Cells(1, 7) = Format(lngDiscr) + " discrepancies and " + Format(lngMiss) + " missing genotypes have been found"

Cells(1, 7).Font.ColorIndex = 3

Cells(1, 7).Font.Bold = True

ActiveWindow.WindowState = xlNormal

MsgBox "The comparison has been done successfully." + vbCr + Chr(13) + Chr(13) + "Please, check , in the TEMP sheet, if there are any discrepancies and correct them in the Consensus Column." + vbCr + Chr(13) + Chr(13) + _

"Keep only ONE result for the transfer in your results file.", vbInformation, "Comparison Completed !"

Else

Do While Cells(IntLoop, 1) <> "" ' Process all the rows in the temp worksheet

StrGeno(1) = Cells(IntLoop, 5)

If StrGeno(1) = "" Then Cells(IntLoop, 7) = "This Reading is missing "

Cells(IntLoop, 3) = GetCell(Cells(IntLoop, 3), IIf(intPlate = 96, 12, 24)) ' Translate the cells from values to grid references

Cells(IntLoop, 8) = ""

IntLoop = IntLoop + 1

Loop

End If

' Save the run report to a spreadsheet only if dble reading is processed

If blnDbleReading = "True" Then

strRunReportFile = Application.GetSaveAsFilename(initialFilename:="RunReport.xls")

If strRunReportFile <> "False" Then

Worksheets("Run Report").Copy

ActiveWorkbook.SaveAs strRunReportFile

ActiveWorkbook.Close

End If

End If

Worksheets("Temp ").Activate ' Display the welcome screen again

Application.ScreenUpdating = True

End Sub

Private Function AnalyseFile(ByVal strPath As String, ByVal intFileNum As Integer, ByVal strResultFileName As String) As Boolean

Dim intFile As Integer ' Used to loop through each file

Dim strFilename As String ' Name of the file being processed

Dim strPlateName As String

Dim strPlateID As String

Dim intFirstWell As Integer ' Row of the first well

Dim intLastWell As Integer ' Row of the last cell

Dim intNumberOfSamples As Integer ' Number of samples in the sheet (96 or 384)

Dim cl As Range ' Cell used for Find operations

Dim intNameCol As Integer

Dim intMarkerCol As Integer

Dim intCallCol As Integer

Dim strMarkerName As String

Dim intReportRow As Integer ' The current row in the report sheet

Dim strAll(1 To 2) As String

Dim IntLoop As Integer ' Generic looping variable

Dim lngHomo1 As Long

Dim lngHomo2 As Long

Dim lngHetero As Long

Dim lngNTC_count As Long

Dim lngUndet_count As Long

Dim strName(400) As String

Dim strWell(400) As String

Dim strSample(400) As String

Dim StrGeno(400) As String

Dim intRow As Integer

Dim sngNTCObs As Single

Dim sngUndertObs As Single

Dim lngN As Long

Dim sngHomo1Obs As Single

Dim sngHomo2Obs As Single

Dim sngHeteroObs As Single

Dim sngP As Single

Dim sngQ As Single

Dim sngHomo1Exp As Single

Dim sngHomo2Exp As Single

Dim sngHeteroExp As Single

Dim sngXsqHomo1 As Single

Dim sngXsqHomo2 As Single

Dim sngXsqHetero As Single

Dim sngXsq As Single

Dim sngHwpVal As Single

Dim intTempRow As Integer ' Row counter for the Temp worksheet

Dim blnDbleReading As Boolean 'Double reading is optional

Dim rg As Range ' Cell used for Find operations

Dim strAlls As String

AnalyseFile = False

intReportRow = 2 + intFileNum ' Get the starting rows for the Report and Temp worksheets

intTempRow = IIf(intFileNum = 1, 3, Worksheets("Temp ").Cells(1, 1).End(xlDown).Row + 1)

'Process the batch of files

#If Mac Then ' Use FileFind on Mac's, FileSearch on PC's

With Application.FileFind

.SearchPath = strPath

#Else

With Application.FileSearch

.LookIn = strPath

.FileType = msoFileTypeAllFiles

#End If

.Execute

For intFile = 1 To .FoundFiles.Count ' Work through each file in batch #1

strFilename = .FoundFiles(intFile)

Workbooks.Open strFilename ' Open the workbook

strPlateName = ActiveWorkbook.Name ' Get the name of the workbook

strPlateID = Left$(Cells(3, 2), 6) ' And the Plate ID

' Locate the first cell after the Well heading

intFirstWell = Columns(1).Find("Well", , xlValues, xlWhole).Row + 1

intLastWell = Cells(intFirstWell, 1).End(xlDown).Row

intNumberOfSamples = intLastWell - intFirstWell + 1

intPlate = IIf(intNumberOfSamples > 96, 384, 96)

'Finds column of samples name (NameC), of markername (MarkerC), of calls (CallC)

intNameCol = Rows(intFirstWell - 1).Find("Sample Name", , xlValues, xlWhole).Column

intMarkerCol = Rows(intFirstWell - 1).Find("Marker Name", , xlValues, xlWhole).Column

intCallCol = Rows(intFirstWell - 1).Find("Call", , xlValues, xlWhole).Column

'Reads markerName and All1 & All2

strMarkerName = Cells(intFirstWell, intMarkerCol)

'strAlls = Right$(strMarkerName, 2)

'strAll(1) = Right$(strAlls, 1)

'strAll(2) = Left$(strAlls, 1)

''''''''''''''''''''''''''Data Storage in "Run Report" sheet ''''''''''''''''''''''''''

'If intFileNum = 2 Then blnDbleReading = "True"

' If blnDbleReading = "True" Then

With Workbooks(strMainWorkbook).Worksheets("Run Report")

If intFileNum = 1 Then .Cells(1, 1) = "Report of Run of " + Str(Date) + " at " + Str(Time)

If IsEmpty(.Cells(intReportRow, 1)) Then

.Cells(intReportRow, 1) = "operator " & intFileNum

.Cells(intReportRow, 2) = strPlateID

.Cells(intReportRow, 3) = strMarkerName

.Cells(intReportRow, 4) = strPath

.Cells(intReportRow, 5) = strPlateName

.Cells(intReportRow, 6) = intNumberOfSamples

End If

End With

' End If

'''''''''''''''''''''''''''''''' Checks the marker name in the results file'''''''''''''''''''''

'''''''''''''''' Gets the All1 and All2 in the results file in order to copy genotype from PlateFile To Temp sheet

With Workbooks(strResultFileName).Worksheets(1)

'Locate the marker in the results file

Set cl = .Rows(1).Find(strMarkerName, , xlValues, xlWhole)

If cl Is Nothing Then

'Reads markerName and All1 & All2 in the TaqMan Input Files

strAlls = Right$(strMarkerName, 2)

strAll(1) = Left$(strAlls, 1)

strAll(2) = Right$(strAlls, 1)

Else

strAll(1) = Left$(.Cells(2, cl.Column), 1) ' We found it so record the allele values

strAll(2) = Right$(.Cells(2, cl.Column), 1)

End If

Set cl = Nothing

End With

''''''''''''''''''''''''''''''''Returns to the plateFile to copy the data''''''''''''''''''''''''''''''''''''''''''''''''''''''''

'Checks whether there are names unber "Sample Name"

If intFileNum = 1 And (Cells(intFirstWell, intNameCol) = "A1" Or Cells(intFirstWell, intNameCol) = "A2") Then

MsgBox "I have not found sample name information in your file." + vbCr + _

"You should load your sample name first!", vbCritical, "SamplesNames?"

Workbooks(strMainWorkbook).Activate

Sheets("Welcome !").Select

Exit Function

End If

'''''''''''''''''''''''''gather info on samples of batch 1''''''''''''''''''''''''''''''''

lngHomo1 = 0

lngHomo2 = 0

lngHetero = 0

lngNTC_count = 0

lngUndet_count = 0

For intRow = intFirstWell To intLastWell

strWell(intRow) = Cells(intRow, 1)

strName(intRow) = Cells(intRow, intNameCol)

StrGeno(intRow) = Cells(intRow, intCallCol)

strSample(intRow) = strName(intRow) & "-" + strPlateID & "-" & strWell(intRow) & "-" & strMarkerName

If StrGeno(intRow) <> "NTC" And StrGeno(intRow) <> "Both" And Right(StrGeno(intRow), 1) = strAll(1) Then

StrGeno(intRow) = strAll(1) + "/" + strAll(1)

lngHomo1 = lngHomo1 + 1

ElseIf StrGeno(intRow) <> "NTC" And StrGeno(intRow) <> "Both" And Right(StrGeno(intRow), 1) = strAll(2) Then

StrGeno(intRow) = strAll(2) + "/" + strAll(2)

lngHomo2 = lngHomo2 + 1

ElseIf StrGeno(intRow) = "Both" Then

StrGeno(intRow) = strAll(1) + "/" + strAll(2)

lngHetero = lngHetero + 1

ElseIf StrGeno(intRow) = "NTC" Then

StrGeno(intRow) = "-"

lngNTC_count = lngNTC_count + 1

ElseIf StrGeno(intRow) = "Undetermined" Then

StrGeno(intRow) = "-"

lngUndet_count = lngUndet_count + 1

End If

Next

With Workbooks(strMainWorkbook).Worksheets("Temp ")

For intRow = intFirstWell To intLastWell

If intFileNum = 1 Then

' Record the results into the temp worksheet

.Cells(intTempRow, 1) = strName(intRow)

.Cells(intTempRow, 2) = strPlateID

.Cells(intTempRow, 3) = strWell(intRow)

.Cells(intTempRow, 4) = strMarkerName

.Cells(intTempRow, 5) = StrGeno(intRow)

.Cells(intTempRow, 8) = strSample(intRow)

intTempRow = intTempRow + 1

Else

' locate the sample in the temp file (put there during file 1 processing)

Set cl = .Columns(8).Find(strSample(intRow), , xlValues, xlWhole, xlByColumns)

If cl Is Nothing Then

.Cells(intTempRow, 1) = strName(intRow)

.Cells(intTempRow, 2) = strPlateID

.Cells(intTempRow, 3) = strWell(intRow)

.Cells(intTempRow, 6) = StrGeno(intRow)

intTempRow = intTempRow + 1

Else

.Cells(cl.Row, 6) = StrGeno(intRow)

End If

Set cl = Nothing

End If

Next 'reference to IntRow

End With

' Statistical Analysis

With Workbooks(strMainWorkbook).Worksheets("Run Report")

sngNTCObs = ((lngNTC_count * 100) / intNumberOfSamples)

sngUndertObs = ((lngUndet_count * 100) / intNumberOfSamples)

lngN = (intNumberOfSamples - lngNTC_count - lngUndet_count)

sngHomo1Obs = ((lngHomo1 * 100) / lngN)

sngHomo2Obs = ((lngHomo2 * 100) / lngN)

sngHeteroObs = ((lngHetero * 100) / lngN)

sngP = ((lngHomo1 * 2) + lngHetero) / (lngN * 2)

sngQ = ((lngHomo2 * 2) + lngHetero) / (lngN * 2)

sngHomo1Exp = (sngP ^ 2) * lngN

sngHomo2Exp = (sngQ ^ 2) * lngN

sngHeteroExp = 2 * sngP * sngQ * lngN

sngXsqHomo1 = ((lngHomo1 - sngHomo1Exp) ^ 2) / sngHomo1Exp

sngXsqHomo2 = ((lngHomo2 - sngHomo2Exp) ^ 2) / sngHomo2Exp

sngXsqHetero = ((lngHetero - sngHeteroExp) ^ 2) / sngHeteroExp

sngXsq = sngXsqHomo1 + sngXsqHomo2 + sngXsqHetero

sngHwpVal = Application.WorksheetFunction.ChiDist(sngXsq, 1)

.Cells(intReportRow, 7) = Format(lngUndet_count) + " (" + Format((Int(10000 * lngUndet_count / intNumberOfSamples)) / 100) + "%) "

.Cells(intReportRow, 8) = Format(lngNTC_count) + " (" + Format((Int(10000 * lngNTC_count / intNumberOfSamples)) / 100) + "%)"

.Cells(intReportRow, 9) = lngN

.Cells(intReportRow, 10) = Format(lngHomo1) + " (" + Format((Int((10000 * lngHomo1) / lngN)) / 100) + "%)"

.Cells(intReportRow, 11) = Format(lngHetero) + " (" + Format((Int(10000 * lngHetero / lngN)) / 100) + "%)"

.Cells(intReportRow, 12) = Format(lngHomo2) + " (" + Format((Int(10000 * lngHomo2 / lngN)) / 100) + "%)"

.Cells(intReportRow, 13) = Format(CInt(sngHomo1Exp)) + " (" + Format((Int(10000 * sngHomo1Exp / lngN)) / 100) + "%)"

.Cells(intReportRow, 14) = Format(CInt(sngHeteroExp)) + " (" + Format((Int(10000 * sngHeteroExp / lngN)) / 100) + "%)"

.Cells(intReportRow, 15) = Format(CInt(sngHomo2Exp)) + " (" + Format((Int(10000 * sngHomo2Exp / lngN)) / 100) + "%)"

.Cells(intReportRow, 16) = sngHwpVal

.Range(.Cells(intReportRow, 1), .Cells(intReportRow, 16)).Font.ColorIndex = 1 + (intFileNum * 2)

.Columns("A:A").EntireColumn.AutoFit

End With 'reference to Run Report

intReportRow = intReportRow + 2

Workbooks(strPlateName).Close SaveChanges:=False

Next

End With

AnalyseFile = True ' Indicate succesful completion

End Function

Private Function GetCell(ByVal intValue As Integer, ByVal intCellsPerRow As Integer) As String

' Purpose: This function translates a cell value to a grid reference.

' it is passed the cell value and the number of cells in each row

Dim intRemainder As Integer

intRemainder = intValue Mod intCellsPerRow

If intRemainder = 0 Then ' Check for the last hole in the row

intRemainder = intCellsPerRow

intValue = intValue - 1

End If

' Start at row A and add on the cell number

GetCell = Chr(Asc("A") + intValue \ intCellsPerRow) & intRemainder

End Function

# Module “Temp2Results”

Option Explicit

Option Compare Text

*' Purpose: This procedure is used to transfer consensus information from the Temp worksheet of the current workbook to the first worksheet of the Results workbook.*

*' The Results workbook is assumed to have been previously opened and its name recorded on the Data worksheet.*

Sub TempToResults()

Dim intRow As Integer ' Used to process each row in the Temp worksheet

Dim intResultsRow As Integer ' Used to locate data in the results file

Dim intReportRow As Integer ' Used to record concesus data into the report file

Dim strSample As String ' Identifier for the sample in the Temp worksheet

Dim strMarkerName As String ' Name of the marker from the Temp worksheet

Dim strResultsFileName As String ' Name of the results file worksheet. This file must already be open

Dim strReportFileName As String ' Name of the report file worksheet.

Dim strName As String ' Sample name

Dim strPlateID As String ' Plate ID from results worksheet

Dim StrGeno As String ' Data from the Results worksheet

Dim strConsensus As String ' Data from the Temp worksheet

Dim strPosition As String ' Position of the sample from the results worksheet

Dim intMarkerCol As Integer ' Column in the Results worksheet that contains the marker

Dim cl As Range ' Range used for various Find operations

Dim blnQC As Boolean ' Indicates if quality control analysis is to be performed

Dim blnDbleReading As Boolean ' Indicates if double reading analysis is to be performed

Dim LastCol As Long ' last column used in the results file

Dim NextCol As Integer 'first free coloumn to store sample text

Dim strAll(1 To 2) As String

Initiate ' Get the OS type and name of the main workbook

strResultsFileName = Worksheets("Data").Cells(4, 2) ' Get the results filename

Sheets("Temp ").Select

' Check for rows without consensus

If Cells(3, 6) <> "" Then

blnDbleReading = True

End If

If Not Columns(7).Find("First", , xlValues, xlPart) Is Nothing And blnDbleReading = True Then

MsgBox "There are some discrepancies in your consensus data" + vbCrLf + _

"Please choose ONE result in the Temp Sheet before transferring data" + vbCrLf + _

" I can not proceed", vbCritical, "DataTransfer"

Else

' Find out if they want to analyse quality control data

blnQC = MsgBox("Do you want to analyse Quality Control data?", vbQuestion + vbYesNo, "Quality Control") = vbYes

If blnQC Then

MsgBox "Please choose where you want to save your report file" + vbCrLf + "and enter its name"

strReportFileName = Application.GetSaveAsFilename(initialFilename:=Left(strResultsFileName, Len(strResultsFileName) - 4) + "_QC_Report.xls")

If strReportFileName <> "False" Then

' Make sure we have an xls extension

If Right$(strReportFileName, 4) <> ".xls" Then strReportFileName = strReportFileName + ".xls"

Workbooks.Add

ActiveWorkbook.SaveAs Filename:=strReportFileName

strReportFileName = ActiveWorkbook.Name ' Get the name of the workbook

' Writes in report file the data of sample set and marker

Cells(1, 1) = "Quality control report generated by TaqMan QC Wizard ® on " + Str(Date) + " at " + Str(Time)

Cells(2, 1) = "Dataset"

Cells(2, 2) = strResultsFileName

Cells(5, 1) = "Discrepancies between first reading and QC data"

Cells(6, 1) = "Sample"

Cells(6, 2) = "Marker"

Cells(6, 3) = "Consensus reading"

Cells(6, 4) = "QC data"

Cells(6, 5) = "Assay Plate"

Cells(6, 6) = "Position in Assay plate"

intReportRow = 7

Rows("7:7").Select

ActiveWindow.FreezePanes = True

Windows(strMainWorkbook).Activate

Else

blnQC = False

End If

End If

' Calculate the sample text for each row in the Results file

Windows(strResultsFileName).Activate

With Workbooks(strResultsFileName).Worksheets(1)

NextCol = Rows(1).End(xlToRight).Column + 1

End With

'first loop in order to look for the marker name

intRow = 3 ' Process each line in the temp worksheet

Windows(strMainWorkbook).Activate

Do While Not IsEmpty(Cells(intRow, 1))

strMarkerName = Cells(intRow, 4)

With Workbooks(strResultsFileName).Worksheets(1)

Set cl = .Rows(1).Find(strMarkerName, , xlValues, xlWhole) ' Make sure we have the marker in the results file

If cl Is Nothing Then 'If not add its name to results file

Windows(strResultsFileName).Activate

Cells(1, NextCol) = strMarkerName

strAll(1) = Mid(strMarkerName, Len(strMarkerName) - 1, 1)

strAll(2) = Right(strMarkerName, 1)

Cells(2, NextCol) = strAll(1) + "/" + strAll(2)

NextCol = NextCol + 1

Windows(strMainWorkbook).Activate

End If

End With

intRow = intRow + 1

Loop

'second loop in order to paste the results

' Calculate the sample text for each row in the Results file

Windows(strResultsFileName).Activate

With Workbooks(strResultsFileName).Worksheets(1)

NextCol = Rows(1).End(xlToRight).Column + 1

.Cells(3, NextCol).Formula = "= A3 & ""-"" & B3 & ""-"" & C3 & D3"

intRow = .UsedRange.Rows.Count

.Range(.Cells(3, NextCol), .Cells(intRow, NextCol)).FillDown

End With

intRow = 3 ' Process each line in the temp worksheet

Windows(strMainWorkbook).Activate

Do While Not IsEmpty(Cells(intRow, 1))

strName = Cells(intRow, 1)

strSample = strName + "-" + Cells(intRow, 2) + "-" + Cells(intRow, 3)

strMarkerName = Cells(intRow, 4)

If blnDbleReading = "True" Then

strConsensus = Cells(intRow, 7)

Else

strConsensus = Cells(intRow, 5)

End If

With Workbooks(strResultsFileName).Worksheets(1)

Set cl = .Rows(1).Find(strMarkerName, , xlValues, xlWhole) ' look for thr column of the marker

intMarkerCol = cl.Column ' record the column corresponding to the marker

Set cl = .Columns(NextCol).Find(strSample, , xlValues, xlWhole) ' Try to find the sample text in column 10

If Not cl Is Nothing Then ' If we found it record the consensus

.Cells(cl.Row, intMarkerCol) = strConsensus

End If

End With

' Analyse quality control

If blnQC Then

With Workbooks(strResultsFileName).Worksheets(1)

If Left$(strName, 3) = "Qc_" Then ' Only process QC lines

strName = Mid$(strName, 4)

Set cl = .Columns(1).Find(strName, , xlValues, xlWhole) ' Check for this sample in the results workbook

If Not cl Is Nothing Then ' If we found the corresponding name

intResultsRow = cl.Row

StrGeno = .Cells(intResultsRow, intMarkerCol)

strPlateID = .Cells(intResultsRow, 2)

strPosition = .Cells(intResultsRow, 3) & .Cells(intResultsRow, 4)

If StrGeno <> strConsensus Then ' If the results are different, record the details

With Workbooks(strReportFileName).Worksheets(1)

.Cells(intReportRow, 1) = strName

.Cells(intReportRow, 2) = strMarkerName

.Cells(intReportRow, 3) = StrGeno

.Cells(intReportRow, 4) = strConsensus

.Cells(intReportRow, 5) = strPlateID

.Cells(intReportRow, 6) = strPosition

End With

intReportRow = intReportRow + 1

End If

End If

End If

End With

End If

intRow = intRow + 1

Loop

Workbooks(strMainWorkbook).Worksheets("Temp ").Select

'LastLine = Columns(1).End(xlDown).Row

'LastCol = Rows(1).End(xlToRight).Column

Range(Cells(3, 1), Cells(Columns(1).End(xlDown).Row, Rows(1).End(xlToRight).Column)).Delete

Range("G1").ClearContents

Workbooks(strMainWorkbook).Worksheets("Run Report").Select

'LastLine = Columns(1).End(xlDown).Row

'LastCol = Rows(1).End(xlToRight).Column

Range(Cells(3, 1), Cells(Columns(1).End(xlDown).Row, Rows(1).End(xlToRight).Column)).Delete

If blnQC Then

With Workbooks(strReportFileName).Worksheets(1)

If intReportRow = 7 Then .Cells(intReportRow, 1) = "Congratulations! No discrepancies found !"

intReportRow = intReportRow + 1

.Cells(intReportRow, 1) = "End of the report"

Workbooks(strReportFileName).Close SaveChanges:=True

End With

End If

End If

Workbooks(strResultsFileName).Worksheets(1).Columns(NextCol).Delete ' Delete the sample values

MsgBox "The TaqMan data transfer in your Results File has been done successfully! ", vbExclamation, "The End ! "

Workbooks(strResultsFileName).Activate

Workbooks(strMainWorkbook).Close SaveChanges:=False

End Sub

# Module “Replace”

Option Explicit

Sub Replace()

## ‘Purpose: this module allows the user to get rid of discrepancies, to replace them by “-“

Dim IntLoop As Integer

Dim StrGeno As String

Dim lngMiss As Long

If MsgBox("Do you want to replace all dicrepancies by '- ' ? ", vbYesNo, "Replace") = vbYes Then

IntLoop = 3

Do While Cells(IntLoop, 7) <> ""

StrGeno = Cells(IntLoop, 7)

If Len(StrGeno) > 3 Then Cells(IntLoop, 7) = "-"

IntLoop = IntLoop + 1

Loop

Else: Exit Sub

End If

'counts the missing genotypes

lngMiss = 0

IntLoop = 3

Do While Cells(IntLoop, 1) <> "" ' Process all the rows in the temp worksheet

If Cells(IntLoop, 7) = "-" Then lngMiss = lngMiss + 1

IntLoop = IntLoop + 1

Loop

Cells(1, 7) = " 0 discrepancies and " + Format(lngMiss) + " missing genotypes have been found"

Cells(1, 7).Font.ColorIndex = 3

Cells(1, 7).Font.Bold = True

ActiveWindow.WindowState = xlNormal

End Sub

# Module “Nucleotides Translation”

Option Explicit

Option Compare Text

Sub Translation()

*' Purpose: This procedure translates allele values from the Results workbook.*

Dim intAll(1 To 2) As Integer ' Allele values

Dim shtTranslation As Worksheet ' Pointer to the Translation worksheet in the results workbook

Dim intRow As Integer ' used to loop through each row in the results worksheet

Dim intMarker As Integer ' Used to loop through each marker in the results worksheet

OpenResultFile.OpenResultFile (False) ' Open the results file (not updating the main worksheet)

Application.ScreenUpdating = False ' Stop updating (prevents screen flicker)

ActiveSheet.UsedRange.Copy ' Copy the data from the first worksheet

Set shtTranslation = Worksheets.Add ' Add a new worksheet to the results workbook

shtTranslation.Name = "Translation" ' Rename it, paste the data and select it

shtTranslation.Paste

shtTranslation.Select

intMarker = 5 ' markers start at column 5

Do While Not IsEmpty(Cells(1, intMarker)) ' Process all markers

If Cells(2, intMarker) <> "-" Then ' Skip - entries

intRow = 3 ' Allele values start at row 3

Do While Not IsEmpty(Cells(intRow, 1)) ' Process all results

intAll(1) = 0

intAll(2) = 0

If Cells(intRow, intMarker) <> "" Then ' Skip blank entries

' Convert the allele values from ACGT to 1234 (- = 0)

intAll(1) = InStr("ACGT", Left$(Cells(intRow, intMarker), 1))

intAll(2) = InStr("ACGT", Right$(Cells(intRow, intMarker), 1))

' Enter the converted values

Cells(intRow, intMarker) = intAll(1) & " " & intAll(2)

End If

intRow = intRow + 1

Loop

intMarker = intMarker + 1

End If

Loop

Set shtTranslation = Nothing ' Get rid of the pointer

Application.ScreenUpdating = True

MsgBox "Translation completed"

End Sub

**7. Macro “Count”**

- Module “Read Me”

Sub GoToInstructions()

'

' GoToInstructions Macro

' Macro recorded 11.3.2003 by Federico

'

'

Sheets("Instructions").Select

End Sub

- Module “Count”

Sub Count()

Dim strResultsFile As String ' the results file to proceed

Dim strResultsFileName As String ' the name of the results file to proceed

'Open the results file to count

strResultsFile = False

MsgBox "Please choose your results file", vbQuestion, "Results File?"

strResultsFile = Application.GetOpenFilename

'Gestion du bouton Cancel

If strResultsFile = "False" Then

If MsgBox("No plates file has been selected. Do you want to open your results file?", vbCritical + vbYesNo, "Open results file?") = vbNo Then

MsgBox "See you next time", vbCritical, "Bye Bye"

Exit Sub

End If

End If

Workbooks.Open strResultsFile

strResultsFileName = ActiveWorkbook.Name

MsgBox "Make sure" + strResultsFileName + "is well formatted !", vbExclamation

ActiveSheet.Select

'Find out how many samples there are in total:

y = 2

samples = 0

5

y = y + 1

If Cells(y, 1) = "" Then GoTo 6

samples = samples + 1

GoTo 5

6

'Clears previous values

Range(Cells(y + 2, 1), Cells(y + 17, 1)).Select

Selection.ClearContents

'Write in the data holders:

Cells((y + 3), 1) = "Total Subjects:"

Cells((y + 4), 1) = "Missing (%):"

Cells((y + 5), 1) = "Total Subjects with Genotypes:"

Cells((y + 7), 1) = "Observed Allele 1 Counts (%):"

Cells((y + 8), 1) = "Observed Allele 2 Counts (%):"

Cells((y + 10), 1) = "Observed Homo 1 Subjects (%):"

Cells((y + 11), 1) = "Observed Hetero Subjects (%):"

Cells((y + 12), 1) = "Observed Homo 2 Subjects (%):"

Cells((y + 14), 1) = "Expected Homo 1 Subjects (%):"

Cells((y + 15), 1) = "Expected Hetero Subjects (%):"

Cells((y + 16), 1) = "Expected Homo 2 Subjects (%):"

Cells((y + 18), 1) = "H-W p-value:"

'Figure out how many polymorphisms we have:

x = 1

polys = 0

10

x = x + 1

If Cells(1, x) = "" Then GoTo 11

polys = polys + 1

GoTo 10

11

'Clears previous values

Range(Cells(y + 2, 2), Cells(y + 17, polys + 1)).Select

Selection.ClearContents

'Start counting:

For count_poly = 2 To polys + 1

If Cells(2, count_poly) = "-" Then GoTo 16

Allele1 = Left(Cells(2, count_poly), 1)

Allele2 = Right(Cells(2, count_poly), 1)

Columns(count_poly).Select

Selection.Replace What:=Allele2 & "/" & Allele1, Replacement:=Allele1 & "/" & Allele2, LookAt:=xlPart, _

SearchOrder:=xlByRows, MatchCase:=False

A1 = 0

A2 = 0

H1 = 0

HT = 0

H2 = 0

xsq = 0

xsqH1 = 0

xsqHT = 0

xsqH2 = 0

expH1 = 0

expHT = 0

expH2 = 0

genotypes = 0

missing = 0

For count_sample = 3 To samples + 3

If count_sample = samples + 3 Then GoTo 15

If Cells(count_sample, count_poly) = "-" Then

missing = missing + 1

GoTo 12

End If

If Cells(count_sample, count_poly) = "Redo" Or Cells(count_sample, count_poly) = "?" Or Cells(count_sample, count_poly) = "" Or IsEmpty(Cells(count_sample, count_poly)) Then

Cells(count_sample, count_poly) = "-"

missing = missing + 1

GoTo 12

End If

genotypes = genotypes + 1

If Right(Cells(count_sample, count_poly), 1) = Allele1 Then A1 = A1 + 1

If Right(Cells(count_sample, count_poly), 1) = Allele2 Then A2 = A2 + 1

If Left(Cells(count_sample, count_poly), 1) = Allele1 Then A1 = A1 + 1

If Left(Cells(count_sample, count_poly), 1) = Allele2 Then A2 = A2 + 1

If Cells(count_sample, count_poly) = Allele1 & "/" & Allele1 Then H1 = H1 + 1

If Cells(count_sample, count_poly) = Allele1 & "/" & Allele2 Then HT = HT + 1

If Cells(count_sample, count_poly) = Allele2 & "/" & Allele2 Then H2 = H2 + 1

12 Next count_sample

15

Cells((y + 3), count_poly) = samples

Cells((y + 4), count_poly) = Format(missing) + " (" + Format((Int(10000 * missing / samples)) / 100) + "%)"

Cells((y + 5), count_poly) = genotypes

Cells((y + 7), count_poly) = Format(A1) + " (" + Format((Int(10000 * A1 / (genotypes * 2))) / 100) + "%)"

Cells((y + 8), count_poly) = Format(A2) + " (" + Format((Int(10000 * A2 / (genotypes * 2))) / 100) + "%)"

Cells((y + 10), count_poly) = Format(H1) + " (" + Format((Int(10000 * H1 / (genotypes))) / 100) + "%)"

Cells((y + 11), count_poly) = Format(HT) + " (" + Format((Int(10000 * HT / (genotypes))) / 100) + "%)"

Cells((y + 12), count_poly) = Format(H2) + " (" + Format((Int(10000 * H2 / (genotypes))) / 100) + "%)"

'Calculate expected genotype frequencies

'If there are zero, move on...

If Cells((y + 3), count_poly) = 0 Then GoTo 16

expH1 = (A1 / (genotypes * 2)) ^ 2 * genotypes

expHT = 2 * (A1 / (genotypes * 2)) * (A2 / (genotypes * 2)) * genotypes

expH2 = (A2 / (genotypes * 2)) ^ 2 * genotypes

If expHT < 0.0001 Then GoTo 16

xsqH1 = (((H1) - expH1) ^ 2) / expH1

xsqHT = ((((HT) - expHT) ^ 2) / expHT)

xsqH2 = ((((H2) - expH2) ^ 2) / expH2)

xsq = xsqH1 + xsqHT + xsqH2

hwpval = Application.WorksheetFunction.ChiDist(xsq, 1)

Cells((y + 14), count_poly) = Format(CInt(expH1)) + " (" + Format((Int(10000 * expH1 / (genotypes))) / 100) + "%)"

Cells((y + 15), count_poly) = Format(CInt(expHT)) + " (" + Format((Int(10000 * expHT / (genotypes))) / 100) + "%)"

Cells((y + 16), count_poly) = Format(CInt(expH2)) + " (" + Format((Int(10000 * expH2 / (genotypes))) / 100) + "%)"

Cells((y + 18), count_poly) = hwpval

16

Next count_poly

'Final formatting

Range(Cells(y + 3, 2), Cells(y + 18, polys + 1)).Select

Selection.HorizontalAlignment = xlCenter

Columns("A:A").EntireColumn.AutoFit

Range(Cells(y + 3, 1), Cells(y + 18, 1)).Select

Workbooks("Count.xls").Close SaveChanges:=False

End Sub
